# Intestinal fatty acid-binding protein and gut permeability responses to exercise

**DOI:** 10.1007/s00421-017-3582-4

**Published:** 2017-03-13

**Authors:** Daniel S. March, Tania Marchbank, Raymond J. Playford, Arwel W. Jones, Rhys Thatcher, Glen Davison

**Affiliations:** 1 Department of Infection, Immunity and Inflammation, College of Medicine, Biological Sciences and Psychology, University of Leicester, Leicester, UK; 2 Centre for Immunobiology, Blizard Institute, Barts and The London School of Medicine, Queen Mary University of London, London, UK; 3 Peninsula Medical School, Plymouth University, The John Bull Building, Tamar Science Park, Research Way, Plymouth, UK; 4 Lincoln Institute for Health, University of Lincoln, Lincoln, UK; 5 Institute of Biological, Environmental and Rural Sciences, Aberystwyth University, Aberystwyth, UK; 6 Endurance Research Group, School of Sport and Exercise Sciences, University of Kent, Medway Campus, Chatham Maritime, ME4 4AG UK

**Keywords:** Strenuous exercise, Intestinal permeability, Core temperature, Bovine colostrum, Cell damage, Cellular injury, Urinary *L/R*

## Abstract

**Purpose:**

Intestinal cell damage due to physiological stressors (e.g. heat, oxidative, hypoperfusion/ischaemic) may contribute to increased intestinal permeability. The aim of this study was to assess changes in plasma intestinal fatty acid-binding protein (I-FABP) in response to exercise (with bovine colostrum supplementation, Col, positive control) and compare this to intestinal barrier integrity/permeability (5 h urinary lactulose/rhamnose ratio, *L/R*).

**Methods:**

In a double-blind, placebo-controlled, crossover design, 18 males completed two experimental arms (14 days of 20 g/day supplementation with Col or placebo, Plac). For each arm participants performed two baseline (resting) intestinal permeability assessments (*L/R*) pre-supplementation and one post-exercise following supplementation. Blood samples were collected pre- and post-exercise to determine I-FABP concentration.

**Results:**

Two-way repeated measures ANOVA revealed an arm × time interaction for *L/R* and I-FABP (*P* < 0.001). Post hoc analyses showed urinary *L/R* increased post-exercise in Plac (273% of pre, *P* < 0.001) and Col (148% of pre, *P* < 0.001) with post-exercise values significantly lower with Col (*P* < 0.001). Plasma I-FABP increased post-exercise in Plac (191% of pre-exercise, *P* = 0.002) but not in the Col arm (107%, *P* = 0.862) with post-exercise values significantly lower with Col (*P* = 0.013). Correlations between the increase in I-FABP and *L/R* were evident for visit one (*P* = 0.044) but not visit two (*P* = 0.200) although overall plots/patterns do appear similar for each.

**Conclusion:**

These findings suggest that exercise-induced intestinal cellular damage/injury is partly implicated in changes in permeability but other factors must also contribute.

## Introduction

Intestinal cellular injury may occur in response to exercise due to the effects of various physiological stressors (e.g. heat stress, hypoperfusion/ischaemic stress, oxidative stress, mechanical damage), which may contribute to increased intestinal barrier disturbances and gastrointestinal complications (de Oliveira et al. 2014; Jeukendrup et al. 2000; Pires et al. 2016; van Wijck et al. 2012a). Furthermore, cellular injury, intestinal permeability changes and subsequent endotoxaemia can lead to other serious systemic complications (Davison 2013; Derikx et al. 2007). Although intestinal hypoperfusion/ischaemia, intestinal cellular injury/damage and permeability changes are rare in young healthy individuals they can occur in physiological stress situations such as strenuous exercise (ter Steege et al. 2012; van Wijck et al. 2011). Intestinal hypoperfusion, ischaemia–reperfusion (I–R) and increases in core temperature during (or shortly after) strenuous exercise are primary pathways contributing to intestinal injury and changes in permeability (Davison et al. 2016; Marchbank et al. 2011; Pals et al. 1997; ter Steege et al. 2012; van Wijck et al. 2011, 2012a; Zuhl et al. 2014).

Measurement of the urinary excretion ratio between lactulose and rhamnose (*L/R*) is an accurate, repeatable, non-invasive method of assessing intestinal permeability. It does, however, have some limitations. This method requires ingestion of the probes and a period of fasted urine collection. Furthermore, analysis of urinary *L/R* can be complex, time consuming and the equipment required is not widely available (Miki et al. 1996). Another commonly employed marker is circulating bacterial lipopolysaccharide, LPS (Bosenberg et al. 1988; Brock-Utne et al. 1988; Camus et al. 1997; Jeukendrup et al. 2000). However, this is not a direct measure but rather is a potential consequence or outcome of increased permeability, and it lacks standardisation (i.e. levels may vary/not be standardised within the gut). There are also issues pertaining to the collection and analysis of blood samples in addition to the detection of LPS further suggesting that the use of this marker to assess intestinal permeability changes and/or intestinal cell injury is less than ideal (Camus et al. 1998). For these reasons, and since damage or injury to the intestinal epithelial cells is a key pathway implicated in increased permeability, markers of enterocyte damage or injury as a result of strenuous exercise are of value and desirable. One such marker that has received recent attention and has been suggested as a valid marker of intestinal cell injury or damage following exercise in humans is human Intestinal Fatty Acid-Binding Protein (I-FABP) (Morrison et al. 2014; van Wijck et al. 2011, 2012a, b, 2013, 2014). I-FABP is a 15 kDa cytosolic protein which plays a role in the cellular uptake and metabolism of fatty acids (Ockner and Manning 1974). It is present in the mature enterocytes of the small intestinal villi and is released as soon as cell membrane integrity is compromised and subsequently appears in the circulation following enterocyte injury (half-life 11 min) (Thuijls et al. 2011). Therefore, in young healthy individuals plasma I-FABP following strenuous exercise can be an acute and sensitive measure of intestinal cellular injury. A previous investigation reported a significant increase in plasma I-FABP, paralleled by a significant increase in plasma *L/R* (van Wijck et al. 2011). In a subsequent study (van Wijck et al. 2012b), the same group reported a significant increase in plasma I-FABP which was also paralleled with an increase in urinary *L/R* (5 h) although no direct statistical comparison was made between the increase in permeability following exercise and baseline (van Wijck et al. 2012b). Therefore, it is warranted to investigate whether increases in plasma I-FABP (a marker of intestinal cellular damage) are mirrored by subsequent changes in permeability (measured by urinary *L/R*) using a consistent, validated and reliable method of measurement (Davison et al. 2016; Mahmood et al. 2007; Marchbank et al. 2008, 2011; Playford et al. 2001) and an exercise protocol that is known to increase intestinal permeability (20 min of treadmill running at 80% ${\dot V}$O_2peak_) (Davison et al. 2016; Marchbank et al. 2011).

To further explore the possible link between intestinal cell injury (I-FABP) and permeability (urinary *L/R*), it will be advantageous to assess changes in both plasma I-FABP and urinary *L/R* in response to exercise but also with a positive control known to reduce the increase in permeability (*L/R*) to determine whether similar blunting is concurrently evident for both measures. Fourteen days of oral supplementation with bovine colostrum (Col) has previously been shown to blunt exercise (and other stressor)-induced increases in intestinal permeability (Davison et al. 2016; Marchbank et al. 2011; Playford et al. 2001), hence can serve as a positive control.

Therefore, the purpose of this study was: (1) to determine whether disturbances in the intestinal barrier (increased permeability) using a well-established measurement of permeability (*L/R*) and a relevant exercise protocol are paralleled by changes in a marker of enterocyte damage/injury, plasma I-FABP. (2) To determine whether a positive control (Col), which is known to blunt exercise-induced changes in permeability, can also reduce post-exercise increases in plasma I-FABP. It was hypothesised that changes in permeability would be paralleled by increases in plasma I-FABP (as a marker of enterocyte injury) and that both would be blunted by 14 days of oral Col supplementation (as a positive control).

## Methods

### Ethical approval

The study was conducted in accordance with the Declaration of Helsinki and approved by the Aberystwyth University Research Ethics Committee. All participants were informed both verbally and in writing of the nature and risks of the experimental procedures after which written consent was obtained at least 7 days prior to the preliminary visits.

### Sample size

Based on a pilot study with six participants, we calculated (G*Power version 3.1.9.1, Kiel, Germany) that *n* = 14 was required to detect an arm × time interaction (Pilot data: Plac post-ex 217% of pre-ex vs Col 131%, effect size 0.816) with 95% power and alpha 0.05. We, therefore, recruited a total of 18 participants to account for a drop-out rate of ~20% (although we had no drop-outs on this occasion; hence, *n* = 18 for this study).

### Participants

Eighteen healthy male participants who were all regular exercisers participated in this study (Mean ± SD: age 26 ± 5 years; body mass 77.0 ± 9.8 kg; height 177 ± 8 cm; body mass index 24.5 ± 1.8 ${\dot V}$O_2peak_ 56.3 ± 6.5 mL·kg^−1^·min^−1^; peak speed in incremental exercise test 18.2 ± 1.4 km·h^−1^; running speed at 80% ${\dot V}$O_2peak_ 13.0 ± 1.3 km·h^−1^).

### Inclusion/exclusion criteria

Inclusion criteria were: age 18–45 years, free of illness symptoms for 4 weeks prior to written consent, and no non-steroidal anti-inflammatory drug consumption or other use (e.g. via over the counter creams) for 4 weeks prior to written consent. Individuals were unable to partake if they were currently a smoker, had any history of gastrointestinal disorder or surgery, or were allergic or intolerant to dairy products, or suffered from dizziness or nausea at the sight of needles and/or blood. Subjects were also excluded if they were not able to complete an exercise test that required maximum effort or had recent history (<6 months) of supplement use.

### Bovine colostrum

The Col was supplied by Neovite UK as used in a previous study from our laboratory (Marchbank et al. 2011). The approximate energy content was 311.5 kJ (74.4 kcal) per 20 g (i.e. 80% protein, 9% carbohydrate (lactose), 1.8% fat). The placebo (Plac) used in place of Col was an isoenergetic and isomacronutrient milk protein concentrate and was indistinguishable in terms of appearance and taste from the Col powder.

### Study design and nutritional intervention (positive control)

**Fig. 1:**
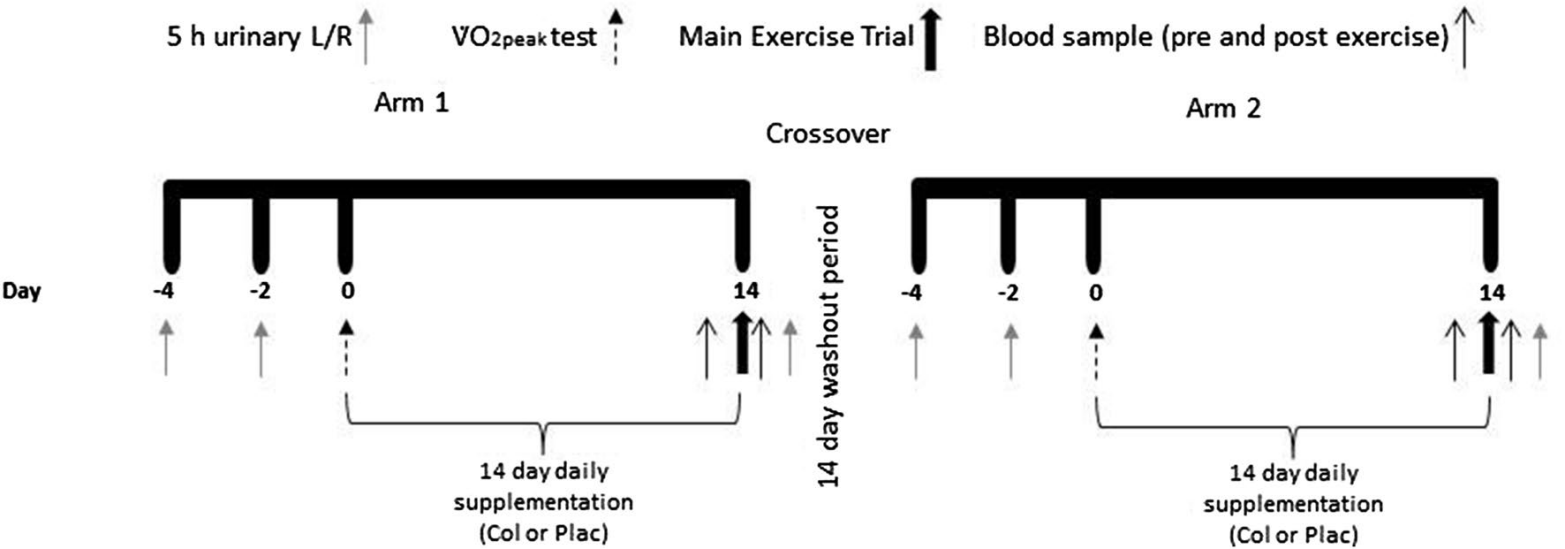
Schematic of study design. Each participant took part in a double-blind, placebo-controlled, randomised, crossover study. Participants received oral supplementation with 20 g·day^−1^ of either Plac or Col (in a randomised crossover design) for 14 days with a 14 days washout period between study arms. After the 14 days washout these procedures were repeated with participants supplementing with the opposite test substance (Plac or Col)

In this double-blind, placebo-controlled, randomised, crossover study, participants were required to undertake exercise under two conditions (study arms): Plac supplementation or Col with a minimum washout period of 14 days between arms (Fig. 1). For each arm, participants were required to visit the laboratory on four occasions over an approximate 3-week period. The four visits included three preliminary assessments (two baseline intestinal permeability assessments by 5 h urinary *L/R* and one determination of running speed at 80% ${\dot V}$O_2peak_) and one main exercise visit. Intestinal permeability assessments (Baseline 1 and Baseline 2) were performed 2 days apart followed by the determination of running speed at 80% ${\dot V}$O_2peak_ in a separate visit at least a further 2 days later. Following this, participants supplemented with 20 g·day^−1^ (10 g prior to morning and evening meal) of either Col or Plac for 14 days, which was administered in a randomised crossover fashion using an online randomisation tool (http://www.randomization.com). Participants then performed the main exercise visit at the end of the supplementation period (14 days later). Intestinal permeability assessments were again performed immediately following completion of exercise (Post-Ex). Supplements were supplied in sealed tubes labelled as ‘A’ and ‘B’ by an independent investigator who was not involved in the data collection or analysis. Investigators were unblinded following the completion of data collection and analysis. Participants completed a 24-h food diary on the day before the first exercise visit and repeated this diet for the subsequent exercise visit in the opposing arm. Participants were instructed to avoid alcohol consumption and strenuous activity or exercise for 2 days before each preliminary and main exercise visit. No non-steroidal anti-inflammatory drug consumption or other use (e.g. via over the counter creams) was allowed for 4 weeks prior to the study and for the duration of the study.

### Intestinal permeability assessment

Permeability was assessed using our previously published protocol, equipment, and methods (Playford et al. 2001). Following an overnight fast, participants were required to empty their bladder and then drink a standardised sugar solution containing 5 g of lactulose, 2 g of mannitol, and 1 g of rhamnose in a total of 450 mL of water (note: mannitol was also included only to maintain consistency with our previous studies, including osmolality, e.g. (Marchbank et al. 2011), calculated osmolality 69 mOsmol/kg). Participants were then allowed ad libitum intake of fluid to ensure adequate urine output. They remained rested and fasted during the collection period. Urine was collected over the next 5 h (after ingestion of the sugar solution) and total volume recorded. Aliquots were centrifuged at 1500×*g* for 10 min at 4 °C to remove gross debris and the supernatant was frozen at −80 °C for later analysis. The various sugars were separated using high-performance liquid chromatography (HPLC) and quantitated using a pulse amphometric detector as previously described (Marchbank et al. 2011). Urinary *L/R* are presented as area under the chromatograph curve rather than percentage urinary excretion ratios as we have previously shown (Marchbank et al. 2011) that expressing them either way unsurprisingly gives equivalent results. The intra-assay variation was 2.3% for duplicate measurements performed in the same participants.

### Preliminary visits

Participants reported to the laboratory and body mass and stature were recorded. Body mass was recorded using laboratory scales (Seca 899, Seca GmbH & Co, Hamburg, Germany) with the participants unshod and wearing the same clothing as during the main exercise visits (typically lightweight shorts and t-shirt). Following this, participants completed a graded exercise treadmill test (Woodway Ergo-Line Treadmill PPS55i Med, Woodway GmbH, Weil am Rhein, Germany) to voluntary exhaustion with the grade set at 1% (Jones and Doust 1996), as previously reported (Carter et al. 2000).

### Main exercise visit

Following an overnight fast, participants reported to the laboratory at 07:00, and confirmed verbally that they had adhered to the supplement intake instructions and other study requirements. Participants were then asked to remain seated for 10 min before a blood sample was drawn (Pre-Ex) (less than 30 s of stasis) from the antecubital vein using a 21-gauge precision needle (Becton–Dickinson, Oxford, UK) and collected into vacutainer tubes (6 mL into K_2_EDTA-treated tube and 6 mL into a heparin-treated tube, BD Vacutainer Systems, Plymouth, UK). A rectal thermistor (Grant Instruments, Cambridge, England) was self-positioned by each participant 10 cm beyond the anal sphincter to enable core temperature measurement and a short-range heart rate telemetry band was fitted (Polar S610i, Polar Electro Oy, Tampere, Finland). Following this, participants performed a 20-min run at a constant speed equivalent to 80% ${\dot V}$O_2peak_ on a motorised treadmill with a 1% grade (Jones and Doust 1996) in temperate conditions (22.1 ± 1.7 °C, and 37 ± 8% relative humidity). Heart rate (HR), and rating of perceived exertion (RPE) (Borg 1970) were recorded (every 5 min) and expired gas was collected and analysed (Jaegar Oxycon Pro, Jaegar, Hoechberg, Germany) every 5 min during the visit. Core temperature was recorded every 2 min and then again 5 min following the cessation of exercise (the change between pre-exercise and 5 min post was calculated). The mean of the physiological responses were calculated to ensure a similar physical stress was imposed between arms. After completing the run participants were immediately seated and a further venous blood sample was obtained (Post-Ex) (no longer than 3 min following the cessation of exercise). Participants then emptied their bladder before consuming the intestinal permeability test solution and commencing with a 5 h urine collection to assess intestinal permeability (Post-Ex).

### Blood analysis

Haemoglobin (Hb) was determined on blood samples using an automated haematology analyser (ABX Pentra 60C+, Horiba Medical, Montpellier, France). Blood lactate and blood glucose were determined using an automated analyser (YSI 2300 Stat Plus, Yellow Springs, OH, USA), and haematocrit was determined by standard microcentrifugation (Micro Haematocrit Mk5 Centrifuge, Hawkesley, Lancing, UK). Haematocrit and Hb concentration were used to estimate post-exercise changes in blood and plasma volume (compared to the Pre-Ex sample) using the equations of Dill and Costill (1974). The blood sample collected in the K_2_EDTA-treated tube was centrifuged at 1500×*g* for 10 min at 4 °C. The plasma from the K_2_EDTA-treated tube was pipetted into polypropylene microcentrifuge tubes (Eppendorf, Hamburg, Germany) before being stored at −80 °C for later analysis (plasma I-FABP).

### Plasma I-FABP analyses

Plasma I-FABP was determined using the EDTA plasma performed in duplicate via an ELISA kit (Hycult, Biotechnology, Uden, The Netherlands) according to the manufacturer’s instructions. Sterile procedures were observed throughout the assay. Inter-assay CV was 5.9%. Changes in plasma I-FABP following exercise were first reported as absolute change (in pg/ml) (van Wijck et al. 2011, 2012b). More recent reports have presented plasma I-FABP as percentage change, compared to baseline or pre-exercise (van Wijck et al. 2013, 2014), as a result of large inter participant variability in plasma I-FABP. To allow consistency with recent reports we have presented changes in this marker as both percentage and absolute values.

### Statistics

All statistical analyses of data were performed using the Statistical Package for Social Sciences (SPSS for Windows, version 21.0, IBM, New York, USA). All data are presented as mean values ± standard deviation (SD) unless otherwise stated. Data were checked for normal distribution by observing the *P* value from the Shapiro–Wilk test. Data not normally distributed (plasma I-FABP) were normalised by natural log (base-e) transformation prior to analysis. A paired samples *t* test was performed to determine differences between arm (Col and Plac) for plasma volume change (Pre-Ex to Post-Ex). A two-way repeated measure ANOVA (arm × time) was performed to compare temporal responses between each arm (Col and Plac) for HR, RPE, ${\dot V}$O_2_, blood glucose, blood lactate, core temperature, urinary *L/R* and absolute plasma I-FABP. When a main interaction effect was evident for urinary *L/R* and absolute plasma I-FABP post hoc paired samples *t* tests were performed to compare Pre-Ex (mean baseline for *L/R*) to Post-Ex changes within each arm (Col and Plac) and between each arm (Col and Plac) for the same time points, Pre-Ex (mean baseline for *L/R*) and Post-Ex. A paired samples *t* test was performed to determine differences between arm (Col and Plac) for percentage change in plasma I-FABP (from Pre-Ex). Effect size (ES) was calculated using Cohen’s D for the Post-Ex difference in mean score (Baseline measures were comparable) for urinary *L/R*, and for the Post-Ex difference from Pre-Ex for plasma I-FABP (absolute and percent change), when a main interaction was evident. To determine any associations, the difference between Baseline/Pre-Ex and Post-Ex values for both measures (plasma I-FABP and urinary *L/R*) were compared using Spearman’s correlation (data were not normally distributed and log transformation could not be used due to some negative change values). It is not possible to pool the data for a single analysis as the repeated measures design (each subject undertook both Col and Plac arms) means this would violate the assumption that observations are independent (required for correlation analysis). As such, each visit was analysed separately for correlations. We chose to analyse the subjects’ visit 1 and visit 2 separately to ensure the necessary independence of observations in each analysis but also ensuring that each analysis contained a mixture of Plac and Col conditions, and hence gave a good representation of the true spread of data for each measure within the study. Statistical significance was accepted at *P* < 0.05 level.

### Testing for carry-over effect

Carry-over effects were investigated by performing an independent samples *t* test for the exercise-induced change for the Plac arm only for those subjects who were randomised to receive Plac first against those who received Plac as the second treatment for Urinary *L/R* and Plasma I-FABP. Paired samples *t* test were used to compare mean Baseline values for Urinary *L/R* between arms (Col and Plac) for those participants who were randomised to receive Col during the first arm, and for Pre-Ex plasma I-FABP concentrations between arms for participants who received Plac during the first arm.

## Results

### Carry-over effect

There was no significant difference for the exercise-induced change in Urinary *L/R* (*P* = 0.937) or plasma I-FABP (*P* = 0.951) in the Plac arm in subjects who were randomised to Plac first against those who received Plac second (suggesting no carryover effects, for example those who had Col first still subsequently responded similarly in the Plac arm to those who performed the Plac arm first). In participants who received Col during the first arm, there was no significant difference between Baseline Urinary *L/R* (*P* = 0.316) between arms (Col and Plac) showing that following 14 days of ‘wash out’ Urinary *L/R* returned to pre-supplementation levels. For those who received Plac as the first treatment there was no difference between Pre-Ex plasma I-FABP (*P* = 0.784) concentrations between arms, showing that 14 days of Col supplementation did not change Pre-Ex plasma I-FABP concentrations.

### Physiological variables

The response of physiological variables (HR, ${\dot V}$O_2_, RPE, blood glucose and blood lactate) to exercise was similar between arms (Col and Plac) (Table 1). A similar pattern of plasma volume change was observed from Pre-Ex between arms: Plac; Post-Ex (−8.9 ± 4.3%) and Col; Post-Ex (−8.3 ± 4.7%), with no significant difference between arms (*P* = 0.521), and so no parameters were corrected for plasma volume changes.

**Table 1:** Physiological and perceptual responses to exercise trials

	Pre-Ex	5 min	10 min	15 min	20 min	Post-Ex	ANOVA *P* values (arm; time; arm × time)
HR (bpm)							
Plac	73 ± 10	161 ± 10*	169 ± 9*	174 ± 10*	177 ± 10*		0.979; <0.001; 0.399
Col	72 ± 9	161 ± 7*	168 ± 7*	174 ± 7*	177 ± 7*		
${\dot V}$O_2_ (L·min^−1^)							
Plac		3.60 ± 0.56	3.75 ± 0.64*	3.75 ± 0.60*	3.82 ± 0.62*		0.131; <0.001; 0.445
Col		3.55 ± 0.55	3.65 ± 0.61*	3.65 ± 0.62*	3.68 ± 0.59*		
RPE							
Plac		12 ± 2	13 ± 2*	15 ± 1*	16 ± 2*		0.310; <0.001; 0.824
Col		12 ± 2	13 ± 2*	14 ± 2*	15 ± 2*		
Blood glucose (mmol^−1^)							
Plac	4.30 ± 0.29					5.27 ± 1.35*	0.293; 0.002; 0.950
Col	4.23 ± 0.45					5.22 ± 1.17*	
Blood lactate (mmol^−1^)							
Plac	0.65 ± 0.23					3.30 ± 1.62*	0.288; <0.001; 0.190
Col	0.68 ± 0.27					3.07 ± 1.36*	

*Significantly increased (*P* < 0.001) compared to 1st measured time point (Pre-Ex or 5 min)

### Core temperature

The increase for core temperature was similar in Plac and Col arms (ANOVA effect of arm *P* = 0.364; effect of time *P* < 0.001; and arm × time interaction *P* = 0.892). The mean increase for core temperature was 1.5 ± 0.3 °C and 1.5 ± 0.3 °C for Plac and Col arms, respectively (Plac 36.9 ± 0.3 °C to 38.4 ± 0.4 °C; Col 36.8 ± 0.3 °C to 38.3 ± 0.4 °C).

### Urinary *L/R*

**Fig. 2:**
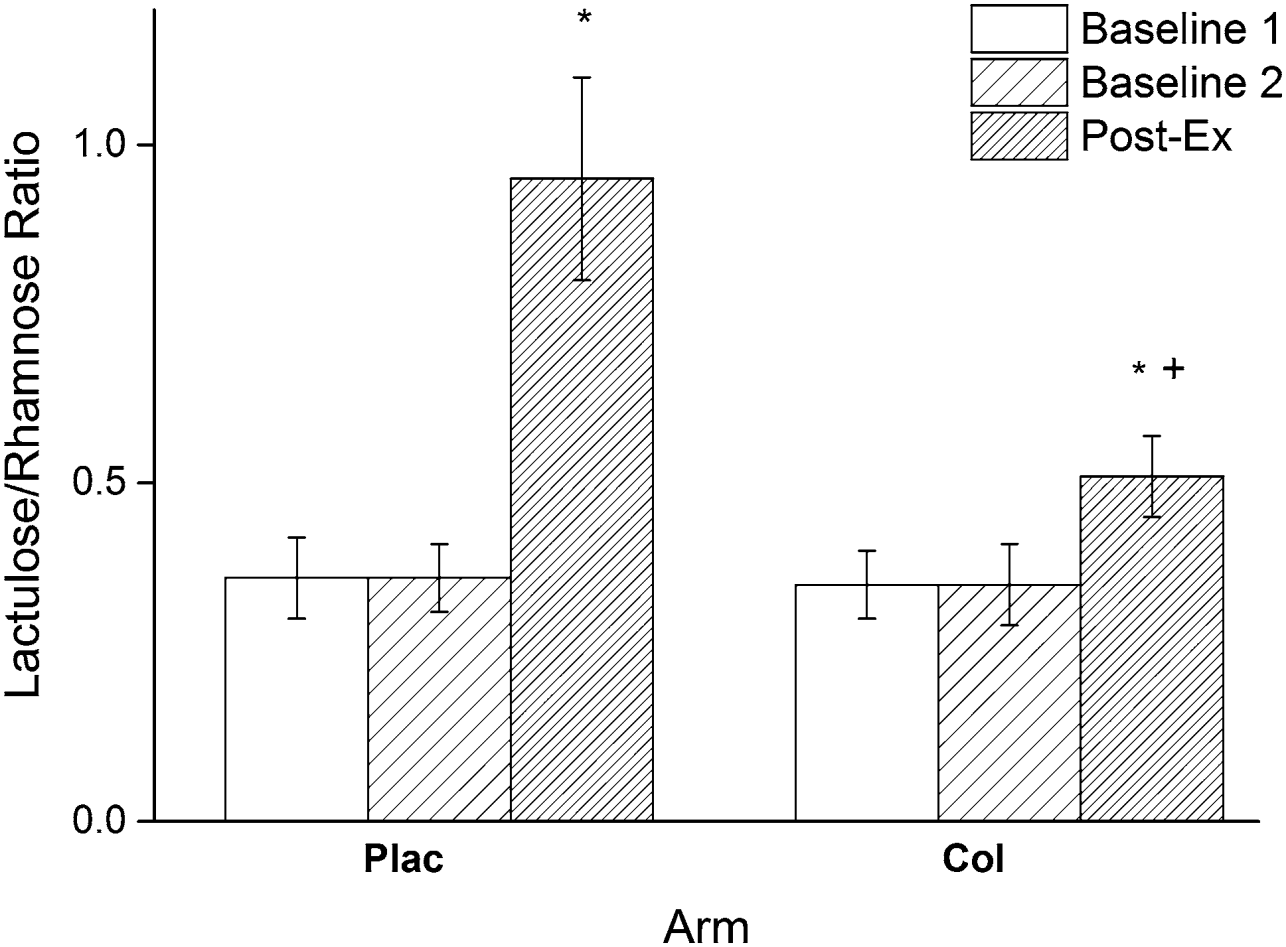
Urinary Lactulose/Rhamnose ratios for Plac and Col arms (mean and SD). *Significantly different from Baseline 1 and Baseline 2 (*P* < 0.001). ^+^Significantly different from Plac at same time point (*P* < 0.001)

Baseline *L/R* values were similar for each arm: Plac; Baseline 1 (0.35 ± 0.06), Baseline 2 (0.35 ± 0.05), Col; Baseline 1 (0.34 ± 0.05), Baseline 2 (0.34 ± 0.06) (Fig. 2). The two-way ANOVA showed that there was a significant main effect of arm (*P* < 0.001), and a significant main effect of time (*P* < 0.001) for urinary *L/R* (5 h). In addition, there was a significant arm × time interaction (*P* < 0.001). Post hoc analysis showed no significant difference between urinary *L/R* Baseline values for each arm (*P* = 0.175). There was a significant increase in urinary *L/R* in both the Plac (273% increase, *P* < 0.001), and Col arms (148% increase, *P* < 0.001). However, there was also a significant difference between Plac and Col arms at Post-Ex (with a large effect size, ES = 4.05, *P* < 0.001, for the change between Pre-Ex and Post-Ex values between arms) showing that the interaction is due to significant blunting of the post-exercise increase by Col (Fig. 2).

### Plasma I-FABP

**Fig. 3:**
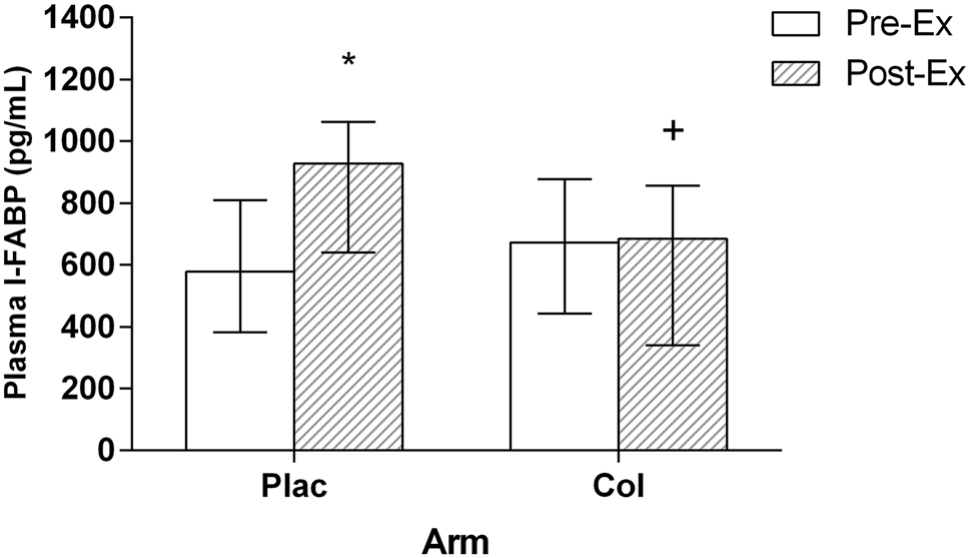
Absolute plasma I-FABP concentration (median and interquartile range). *Significant increase compared to Pre-Ex (*P* = 0.002). ^+^Significantly different from Plac at same time point (*P* = 0.013)

For absolute plasma I-FABP concentration there was no main effect of time (*P* = 0.511). There was a significant main effect of arm (*P* = 0.029), and arm × time interaction effect (*P* = 0.001). Post hoc analysis showed that there was no significant difference between Pre-Ex absolute plasma I-FABP concentrations for each arm (*P* = 0.276). Absolute plasma I-FABP concentration [median and (interquartile range)] significantly increased from Pre-Ex [578 (399) pg/mL] to Post-Ex [928 (382) pg/mL] in the Plac arm (*P* = 0.002); however, this was significantly blunted in the Col arm [Pre-Ex; 672 (394) pg/mL, Post-Ex; 684 (481) pg/mL, *P* = 0.862] (Fig. 3). In addition, there was a significant difference between arms at Post-Ex for absolute plasma I-FABP (*P* = 0.013). There was a large ES (1.05) for the change between Pre-Ex and Post-Ex values between arms. Plasma I-FABP when analysed for percentage change significantly increased from Pre-Ex to Post-Ex in the Plac arm (191 ± 144% increase); however, this was significantly blunted in the Col arm (107 ± 52% increase) (ES = 0.80, *P* = 0.001 compared to increase in Plac).

### Association between Plasma I-FABP and urinary *L/R*

**Fig. 4:**
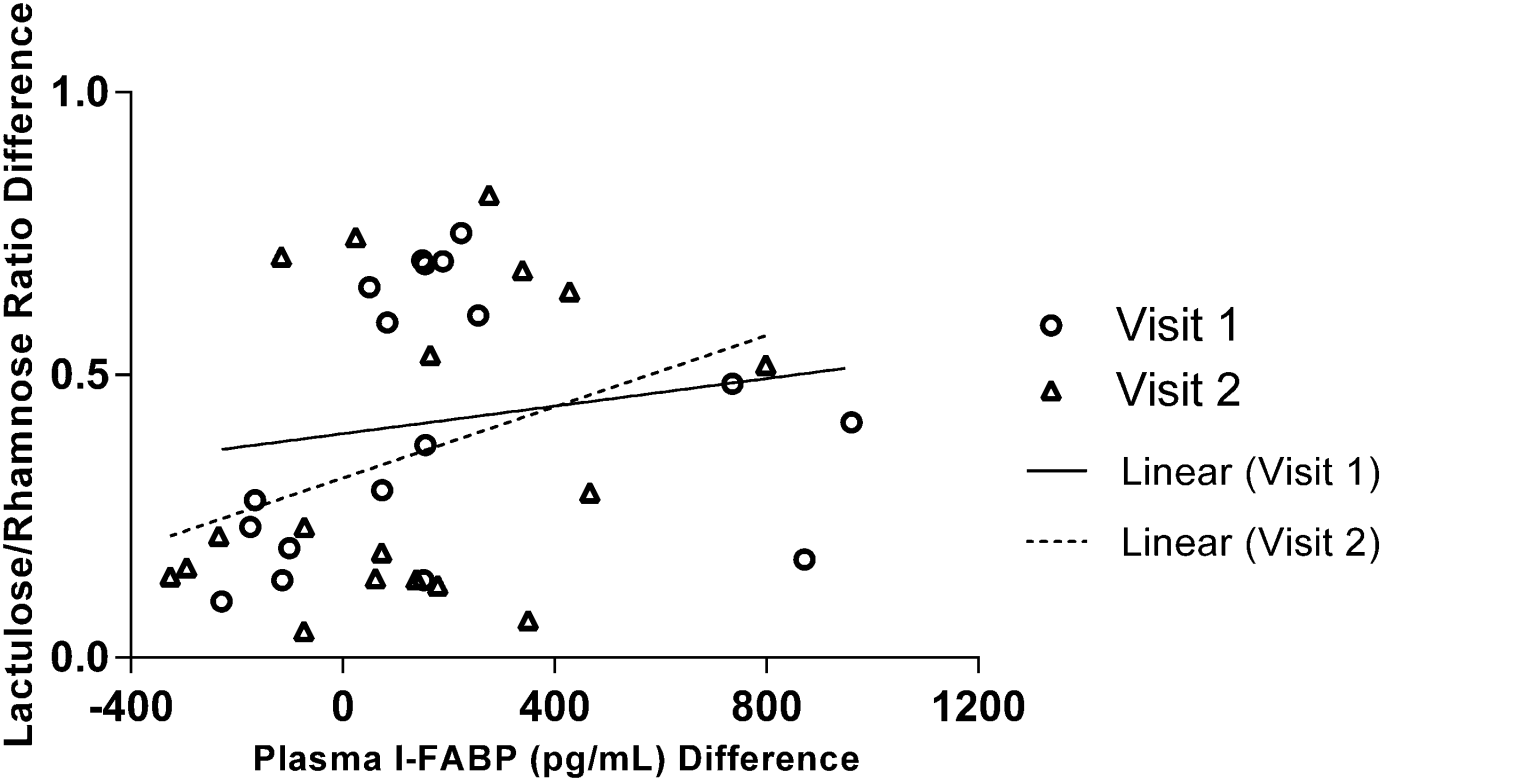
Association between pre-to-post-exercise change in Urinary *L/R* and Plasma I-FABP values. Visit 1 (*P* = 0.034, *R*_s_ = 0.441) and visit 2 (*P* = 0.200, *R*_s_ = 0.212)

The Spearman’s correlation coefficient showed the Post-Ex increase in plasma I-FABP concentration significantly correlated with the Post-Ex increase in urinary *L/R* at visit 1 (*P* = 0.034, *R*_s_ = 0.441) but not at visit 2 (*P* = 0.200, *R*_s_ = 0.212), although plots do suggest similar overall patterns (see Fig. 4).

## Discussion

In a similar manner to previously reported findings (Davison et al. 2016; Marchbank et al. 2011), we observed an increase in intestinal permeability (as measured by urinary *L/R*), following strenuous exercise which was blunted by 14 days of oral Col supplementation (acting as a positive control). The main findings of this study are (1) the exercise-induced change in urinary *L/R* was paralleled by an increase in plasma I-FABP, (2) the exercise-induced increases in urinary *L/R* and plasma I-FABP were both blunted by 14 days of oral Col supplementation. In the current study, presenting plasma I-FABP as absolute concentration or percentage of baseline did not change the pattern of results or overall outcome. The use of the positive control is a novel aspect of the current study which strengthens the suggestion that damage to enterocytes is implicated in permeability increases. It is important to note that plasma I-FABP is not a direct marker of compromised permeability per se, but is a marker of injury or damage to enterocytes and our findings provide evidence that this damage contributes to compromised intestinal barrier function (increased permeability).

The use of urinary *L/R* as a method of assessing intestinal permeability following exercise has previously been shown in this exercise model (Davison et al. 2016; Marchbank et al. 2011). The use of the same mixture of *L/R* (which also includes mannitol to maintain consistency and osmolality) through a series of studies (Davison et al. 2016; Mahmood et al. 2007; Marchbank et al. 2008, 2011; Playford et al. 2001) allows reliable comparisons of the effects of exercise on intestinal barrier permeability to be made. The increase in plasma I-FABP following exercise is in agreement with previously reported observations (Morrison et al. 2014; van Wijck et al. 2011, 2012b, 2013, 2014). During strenuous exercise, there are numerous insults that can affect the intestine, and all of these may contribute to damage and/or increased permeability. This includes heat stress from increased core body temperature, oxidative stress, mechanical damage from body movements (especially during running) and ischaemia/hypoperfusion due to reduced splanchnic blood flow (Davison et al. 2016; de Oliveira et al. 2014; Jeukendrup et al. 2000; Pires et al. 2016; van Wijck et al. 2012a). In a recent systematic review it was reported that increases in core temperature accounted for 63% of the variance in the increase of intestinal permeability seen with exercising (Pires et al. 2016) emphasising the importance of this pathway. Reductions in intestinal perfusion are also known to be an important contributing factor. This occurs as a consequence of the redistribution of blood flow towards exercising muscles (to meet increased oxygen and substrate demand), to the skin for heat dissipation and towards tissues with increased activity such as the heart, lungs and brain (Clausen 1977). The intestines have a low hypoxia tolerance and are particularly sensitive to episodes of hypoperfusion compared to other organs as a result of the vascular architecture of the villi creating a low oxygen state at the tip (Blikslager et al. 2007). This low oxygen environment at the villus tip is exacerbated during exercise resulting in loss of intestinal cell integrity and subsequent release of I-FABP which diffuses through the interstitial space into the circulation upon enterocyte injury (van Wijck et al. 2012a). However, our main aim was to explore the relationship between a marker of enterocyte damage and changes in intestinal permeability, so regardless of the mechanistic cause, we have shown that such damage to cells within the intestinal wall appears to have a key role in the subsequent increase in permeability as detected by increases in *L/R*. As such, assessing acute changes in I-FABP (as a marker of such damage) may be informative and has the advantage of allowing more frequent measurement times during and after exercise. However, the association between the increase in *L/R* and I-FABP was not the strongest and is somewhat variable (Fig. 4). This highlights that although injury may occur to the enterocytes resulting in I-FABP release and this injury contributes to increased permeability it is not the only factor. For example, there are other components within the intestinal barrier (not just the enterocytes), which contribute to maintenance of its barrier function (e.g. tight junctions and related proteins and structures between adjoining enterocytes) and damage/injury to these structures may also compromise barrier function (independently of direct injury to enterocytes, and hence I-FABP release), (Groschwitz and Hogan 2009).

In the present study in the placebo arm plasma I-FABP significantly increased following exercise. An increase from 309 ± 46 pg/mL to 615 ± 118 pg/mL (199% increase) in plasma I-FABP following 60 min of cycling at 70% *W*_max_ has previously been reported (van Wijck et al. 2011), which is similar to the increase reported in the current study. Further studies by the same group have reported approximately 161 and 172% increases in plasma I-FABP immediately following cessation of exercise in the same cycling protocol (van Wijck et al. 2012b, 2014). In the present study we reported an increase in plasma I-FABP after only 20-min exercise, which could be due to greater exercise intensity and consequential greater redistributions of blood flow (ter Steege et al. 2012). Moreover, running compared to cycling elicits larger changes in core temperature, intestinal blood flow (greater musculature activation), and additional injury may be induced by mechanical stress due to the “up and down” movements of the abdominal organs encountered when performing this type of exercise (van Nieuwenhoven et al. 2004). Given that increases in permeability and consequent gastrointestinal complaints are associated with either long-distance strenuous running events (Brock-Utne et al. 1988; Camus et al. 1997) or competition where running accounts for a significant proportion of the race (Bosenberg et al. 1988; Camus et al. 1998; Jeukendrup et al. 2000), the exercise mode (running) employed in the current study seems appropriate in this context.

As previously mentioned I-FABP is primarily expressed in mature enterocytes in the upper half of the villi and is released into the circulation when cell membrane integrity is compromised (Derikx et al. 2007). We have previously shown that Col can increase levels of HSP70, and induce favourable changes in markers of temperature-induced apoptosis (Bax α, Bcl-2 caspase-3 and 9), and tight junction protein phosphorylation (Davison et al. 2016), with the epidermal growth factor present in Col at least partly responsible (Marchbank et al. 2011). Indeed apoptosis is the major cause of cell death during periods of ischaemia and subsequent reperfusion through disruptions between neighbouring cells and the extracellular matrix (Daemen et al. 2002; Ikeda et al. 1998), which high-intensity (above 70% *W*_max_ or 70% ${\dot V}$O_2peak_) exercise induces (van Wijck et al. 2011). The protective cellular mechanisms induced by Col that have been demonstrated in vitro (Davison et al. 2016; Marchbank et al. 2011), along with the findings from the present study showing concurrent reductions in plasma I-FABP and Urinary *L/R* in vivo support the notion that intestinal damage is a key pathway resulting in changes in intestinal permeability during and following exercise. It is important to note that the mechanism of action for Col are likely multifunctional and, therefore, its modulatory effects are probably a result of a number of constituents (Bodammer et al. 2013). It should also be noted that the positive control (Col) did not affect any of the physiological responses in the present study. This shows that the physiological stress/insult was comparable between trials and that the increase in permeability is due to the effects this has on the intestinal barrier (including damage to enterocytes). Furthermore, the blunting seen with Col is likely due to direct protection or repair mechanisms within the intestinal barrier rather than via any effects on physiological responses such as core temperature or splanchnic perfusion. Although we did not measure splanchnic blood flow in the present study it has been shown that reductions in intestinal blood flow during exercise can exacerbate increases in core temperature as heat is not removed from the intestine (Gil et al. 1998; Zuhl et al. 2014), so if there had been any blood flow differences between trials we would expect these to be reflected by different core temperature responses. As the core temperature responses (and other physiological responses) were no different between trials there was likely no difference in blood flow. These points are important in this context because it shows that the physiological stress presented by exercise was consistent between trials and Col acts via protective and/or repair mechanisms on the enterocytes or intestinal barrier in general (as previously mentioned and evidenced by our previous work, e.g. Davison et al. 2016; Marchbank et al. 2011). Together this provides further evidence that exercise-induced injury to enterocytes is a contributing factor in the exercise-induced increases in intestinal permeability.

Changes in plasma I-FABP represent increases in intestinal cellular damage or injury and we have demonstrated a concomitant increase in permeability [as measured by urinary *L/R* (5 h)]. This finding is in contrast with a previous study where plasma I-FABP was not paralleled by changes in intestinal permeability (urinary *L/R*) but a shorter urine collection period (2 h) was used in that study (van Wijck et al. 2014). We used a 5-h urine collection period since longer periods of urine collection (>2 h) may be the preferable method (as a result of difficulties in producing urine in the first 1–2 h post-exercise) for assessing intestinal permeability following exercise with the dual sugar probe method (Davison et al. 2016; Marchbank et al. 2011; Pals et al. 1997; van Wijck et al. 2011). Further explanations could be variances in the administration of the sugar probes or in the amount of lactulose and rhamnose (and subsequent differences in osmolality) used in the mixture. Another important point of note is the degree of hypoperfusion in the latter study (van Wijck et al. 2014), which was lower than previously reported (van Wijck et al. 2011). We reported an average increase in core temperature of 1.5 °C in the present study whilst many previous studies investigating changes in both plasma I-FABP and urinary *L/R* have not measured core temperature (van Wijck et al. 2011, 2012b, 2014). Furthermore, the previous exercise investigations that report significant changes in core temperature do show a concomitant change in permeability during treadmill running (Davison et al. 2016; Marchbank et al. 2011; Pals et al. 1997; Pires et al. 2016; Yeh et al. 2013).

### Limitations

A limitation of the present study is that we did not obtain resting plasma for I-FABP concentration at the same time point as the baseline Urinary *L/R* samples. However, we wanted to minimise the blood sampling demand on participants. Furthermore, we were most interested in the acute pre- to post-exercise increase in plasma I-FABP and are confident (although this is an assumption) that if it was possible to assess *L/R* pre-exercise it would have been equivalent to the baseline values. Indeed, the baseline Urinary *L/R* values are almost identical across the four baseline measurements and comparable to numerous other studies that have shown similar stability using the same methods and mixture of *L/R* (Davison et al. 2016; Mahmood et al. 2007; Marchbank et al. 2008, 2011; Playford et al. 2001); therefore, we believe that it is a reasonable assumption that these baseline *L/R* values would be equivalent to the pre-exercise time point when plasma I-FABP was assessed (and hence baseline to post-exercise *L/R* is a true representation of acute post-exercise changes).

## Conclusions

In summary this is the first study to demonstrate that changes in plasma I-FABP during running exercise are paralleled by increases in intestinal permeability and that both are blunted with 14 days of oral Col supplementation. The inclusion of this positive control arm provides greater confidence in these findings. The findings provide further support that exercise-induced intestinal cellular damage (indicated by increased plasma I-FABP) contributes to changes in intestinal permeability but other factors must also contribute.

## Abbreviations

ANOVA: Analysis of variance
Col: Bovine colostrum
ELISA: Enzyme-linked immunosorbent assay
HPLC: High-performance liquid chromatograohy
HR: Heart rate
I-FABP: Intestinal fatty acid-binding protein
I-R: Ischaemia–reperfusion
LPS: Lipopolysaccharide
Plac: Placebo
RPE: Rating of perceived exertion
*L/R*: Urinary lactulose-to-rhamnose ratio
